# Navigating the landscape of remote patient monitoring in Canada: trends, challenges, and future directions

**DOI:** 10.3389/fdgth.2025.1523401

**Published:** 2025-02-04

**Authors:** Khayreddine Bouabida, Breitner Gomes Chaves, Enoch Anane, Navaal Jagram

**Affiliations:** ^1^ Department of Public Health and Preventive Medicine, Research Center of the Hospital Center of the University of Montreal (CRCHUM), Montréal, QC, Canada; ^2^ École de Santé Publique (ESPUM), Université de Montréal, Montréal, QC, Canada; ^3^ Department of Biomedical Research, St. George’s University School of Medicine, Great River, NY, United States; ^4^ Department of Internal Medicine, The Brooklyn Hospital Center (TBHC), Brooklyn, NY, United States; ^5^ Departement of Community Health Sciences, Université de Sherbrooke, Sherbrooke, QC, Canada

**Keywords:** remote patient monitoring, digital health, virtual care, Canadian healthcare, healthcare technology, AI, perspectives

## Abstract

Remote Patient Monitoring (RPM) has driven significant advancements in Canadian healthcare, especially during the transformative period from 2018 to 2023. This perspective article explores the state of play and examines the current landscape of RPM platforms adopted across Canada, detailing their functionalities and measurable impacts on healthcare outcomes, particularly in chronic disease management and hospital readmission reduction. We explore the regulatory, technical, and operational challenges that RPM faces, including critical issues around data privacy, security, and interoperability, factors essential for sustainable integration. Additionally, this article provides a balanced analysis of RPM's potential for continued growth within Canadian healthcare, highlighting its strengths and limitations in the post-2023 context and offering strategic recommendations to guide its future development. Keywords: Remote Patient Monitoring, Digital Health, Virtual Care, Canadian Healthcare, Healthcare Technology, AI, Perspectives.

## 1 Introduction

The emergence of Remote Patient Monitoring (RPM) has redefined healthcare delivery in Canada, facilitating more comprehensive and accessible care across diverse geographical areas (1–4). Initially accelerated by the urgent demands of the COVID-19 pandemic, RPM has since evolved into a foundational component of Canada's healthcare ecosystem. RPM plays a crucial role in chronic disease management, hospital admission reduction, and enhancing access to care for underserved, rural, and remote communities (1, 4). Given Canada's vast geography and population distribution across urban and remote areas, RPM has become a tailored solution to the nation's unique healthcare challenges (3, 4).

Leading RPM platforms, including TELUS Health, Cloud DX, and McKesson Canada, exemplify the tools designed to monitor various patient health indicators remotely (5–7). These indicators range from vital signs and medication adherence to mental health status, integrating technologies such as wearable devices, telemedicine, and patient engagement interfaces (5–11). The emphasis on proactive, patient-centered healthcare aligns well with the strategic goals of Canadian healthcare institutions, which are under growing pressure from an aging population and the rising prevalence of chronic conditions (12–14). In this context, RPM offers a sustainable pathway to improve healthcare outcomes without adding significant strain to physical healthcare facilities.

However, several challenges limit the seamless adoption and scalability of RPM across Canada. Key concerns include data privacy, regulatory compliance, and technical interoperability, each critical to ensuring secure and effective RPM deployment (15, 16). Compliance with Canadian privacy laws, such as the Personal Information Protection and Electronic Documents Act (PIPEDA) and the Personal Health Information Protection Act (PHIPA), remains central as RPM expands in scope and complexity (15, 16).

This article offers a comprehensive overview of Remote Patient Monitoring (RPM) within the Canadian healthcare system from 2018 to 2023. It explores the state of play and provides a reflective analysis of current trends, the primary RPM platforms in use, and the associated impacts, benefits, and limitations of these technologies. By exploring RPM's evolving role, we present a forward-looking perspective on its potential to enhance healthcare outcomes in Canada. Key strategic areas are identified, including regulatory adaptation, technological advancements, AI integration, and cross-platform interoperability, each essential for ensuring the continued growth and sustained impact of RPM on the healthcare landscape. This perspective aims to guide stakeholders in understanding the current RPM ecosystem and the strategic pathways necessary to maximize its future contributions to Canadian healthcare.

## 2 RPM platforms and functionalities in Canada

A range of RPM platforms have been actively used across Canada, each offering unique functionalities tailored to specific healthcare needs. These platforms combine telemedicine, patient data tracking, remote monitoring, and patient engagement tools, which allow healthcare providers to deliver targeted, timely care, particularly to patients managing chronic conditions. Each platform brings distinct features to the table, reflecting Canada's diverse healthcare landscape and the tailored solutions required to address regional needs.

### 2.1 TELUS health

As a leader in Canada's RPM landscape, TELUS Health provides a comprehensive platform that integrates virtual consultations, remote monitoring, and chronic disease management. With a mission to expand healthcare accessibility, TELUS Health is particularly impactful in facilitating continuous patient-provider communication and supporting remote monitoring, especially in underserved areas. Its platform underscores the importance of seamless, accessible care in areas with limited healthcare resources (5).

### 2.2 Cloud DX

Cloud DX stands out for its real-time data collection and robust patient engagement capabilities. Focused on vital sign monitoring and other key health metrics, Cloud DX supports proactive chronic disease management by allowing healthcare providers to make timely interventions. This capacity to detect health changes early helps reduce hospital readmissions, reinforcing a shift toward more preventive, patient-centered care models (6).

### 2.3 Mckesson Canada

With a strong emphasis on medication management, McKesson Canada's RPM solutions are geared toward supporting patient engagement and adherence to treatment regimens. This focus on medication adherence is especially critical for chronic disease management, where consistency in treatment significantly influences patient outcomes. McKesson's approach highlights the importance of medication management as a core component of effective RPM solutions in Canada (7).

### 2.4 Bayshore healthcare RPM services

Bayshore's RPM services integrate home-based care with remote monitoring, underscoring the continuity of care as patients transition from acute care to a home setting. Real-time monitoring within the home environment reduces the likelihood of hospital readmissions and supports patient autonomy by allowing them to receive ongoing care in a familiar setting (8).

### 2.5 Think research VirtualCare

This platform merges telemedicine with continuous patient monitoring, providing virtual consultations that improve accessibility for patients in remote and rural regions. By reducing the need for in-person visits, Think Research's platform addresses logistical barriers in healthcare delivery, ensuring that patients receive care in a timely and convenient manner (9).

### 2.6 Alayacare and SE health

Both AlayaCare and SE Health incorporate patient engagement tools alongside remote monitoring, fostering a holistic approach to patient care. These platforms support chronic disease management by enabling patient education, self-management, and adherence to care plans. Their commitment to patient empowerment and education is integral to advancing RPM's role in chronic disease care in Canada (10, 11).

These RPM platforms embody the technological progress in healthcare delivery in Canada. Through the integration of telemedicine, real-time data tracking, and continuous monitoring, they enable healthcare providers to deliver enhanced care and improve patient outcomes across diverse settings. Collectively, these platforms reflect an evolving approach to healthcare in Canada, one that values accessibility, prevention, and patient-centered care. The unique capabilities of each platform demonstrate the potential of RPM technologies to address specific healthcare challenges in Canada's urban, rural, and remote communities (13, 14).

## 3 Impacts on healthcare outcomes and economic dynamics

### 3.1 Enhancing chronic care and reducing readmissions

Remote Patient Monitoring (RPM) platforms have demonstrated measurable effectiveness in managing chronic conditions and reducing hospital readmissions. By facilitating continuous monitoring, encouraging patient engagement, and enabling timely interventions, RPM is reshaping chronic care and supporting preventive approaches in Canadian healthcare (1–4, 17–21).

#### 3.1.1 Early detection and timely interventions

Continuous monitoring through RPM allows healthcare providers to identify emerging health issues early, reducing the risk of complications and the need for acute care. Platforms like Cloud DX, known for its real-time data capabilities, empower providers to intervene promptly when a patient's vital signs indicate potential problems. This timely response is instrumental in reducing hospital readmissions, as early detection often prevents minor issues from escalating into serious health events (1–4, 17–21).

#### 3.1.2 Enhanced patient engagement and self-management

RPM fosters a collaborative approach to healthcare by empowering patients to engage actively in their own care. For instance, SE Health's platform integrates patient engagement tools that facilitate self-management, enabling patients to monitor their vital signs, adhere to treatment plans, and access educational resources that inform their health decisions. This active involvement is especially beneficial for chronic disease management, where consistent self-care and adherence to prescribed regimens significantly improve outcomes. By supporting patient autonomy, RPM enhances adherence and ultimately helps mitigate the progression of chronic diseases (1–4, 17–21).

#### 3.1.3 Post-discharge monitoring

Patients discharged from hospitals are often vulnerable to complications that may lead to readmission. RPM provides a valuable tool for continuous post-discharge monitoring, allowing healthcare providers to manage health issues as they arise and ensuring a smoother recovery process. SE Health's RPM platform, for example, supports post-discharge care by tracking patient progress in real-time, which helps to detect and address potential complications before they necessitate readmission. This continuity of care fosters better recovery outcomes and reinforces RPM's role in maintaining patient health beyond the hospital setting (1–4, 17–21).

These outcomes illustrate that RPM not only addresses immediate healthcare needs but also supports long-term health improvements by reducing chronic disease complications and fostering preventive care practices. By promoting early intervention, patient engagement, and post-discharge support, RPM is enhancing the quality of care for Canadian patients and contributing to a healthcare system that is increasingly oriented toward preventive, patient-centered approaches (4, 13, 17–21).

#### 3.1.4 Economic impacts and dynamics

The economic impact of Remote Patient Monitoring (RPM) is an essential consideration for policymakers aiming to scale its adoption across Canada. A comprehensive cost-benefit analysis reveals that RPM has the potential to reduce healthcare costs significantly, primarily by lowering hospital readmission rates, preventing emergency visits, and minimizing the burden on healthcare infrastructure. Studies from the United States and the European Union have highlighted substantial cost savings from RPM adoption, with reductions in hospital admissions and readmission rates being among the most significant contributors to savings. In particular, a systematic review by De Guzman et al. (2022) found that RPM interventions reduced hospital readmissions by 30%–50%, translating into savings of $2,000-$5,000 per patient per year (18). Similarly, in the EU, countries like the UK and Germany have reported cost reductions due to RPM's impact on chronic disease management, with ROI (Return on Investment) rates ranging from 2:1 to 3:1 for various RPM interventions (19).

In Canada, the potential savings from RPM could be even more pronounced, particularly given the country's publicly funded healthcare system and the increasing burden of chronic diseases. According to the Canadian Institute for Health Information (CIHI), the costs associated with managing chronic conditions and hospital readmissions are escalating, with over $6 billion spent annually on hospital readmissions alone (13). Implementing RPM systems can serve as a cost-effective alternative by reducing the need for in-person visits, hospital stays, and acute care interventions, offering a substantial reduction in overall healthcare expenditures.

However, the initial costs of implementing RPM, including platform setup, device procurement, training, and integration with existing systems, must be considered. Despite these upfront investments, the long-term benefits outweigh the costs. The success of RPM in improving healthcare efficiency and patient outcomes, while reducing long-term costs, positions it as an essential strategy for sustainable healthcare in Canada. For policymakers, a robust cost-benefit analysis, inclusive of both direct and indirect savings, will be pivotal in guiding the large-scale adoption of RPM.

## 4 Challenges in RPM implementation

Despite the significant benefits, several challenges hinder the broader adoption and effectiveness of RPM across Canada (4, 13, 17–21).

### 4.1 Data privacy and security

Protecting patient information is paramount in RPM, where sensitive health data is continuously transmitted and stored. Compliance with privacy regulations, including the Personal Information Protection and Electronic Documents Act (PIPEDA) and the Personal Health Information Protection Act (PHIPA), is essential but challenging, especially as data security threats continue to escalate. Many RPM platforms are investing in robust encryption and cybersecurity measures to safeguard patient data; however, privacy concerns remain a substantial barrier to widespread adoption (13, 19–23).

### 4.2 Interoperability with existing systems

The lack of standardization in data-sharing protocols complicates the integration of RPM with Electronic Medical Records (EMRs). Without seamless interoperability, RPM data cannot be efficiently incorporated into clinical decision-making, limiting its contribution to a holistic healthcare framework. Achieving interoperability across diverse healthcare platforms is essential for RPM to enhance continuity of care and facilitate comprehensive patient management (19–23).

### 4.3 Patient usability and digital literacy

Usability remains a significant issue, particularly for older adults and patients with limited digital literacy. Ensuring that RPM platforms are user-friendly and providing ongoing support are critical to increasing patient adoption. Without such measures, RPM could inadvertently exacerbate healthcare disparities, as not all patients are equally equipped to navigate digital tools and benefit from RPM-enabled care (13, 19–24).

### 4.4 Regulatory barriers

The approval process for new RPM technologies can be slow and complex, often delaying innovation and widespread adoption. Streamlining these regulatory procedures could expedite the deployment of new RPM solutions, allowing for more timely and effective healthcare interventions. Reducing these regulatory hurdles would enable RPM to respond more dynamically to healthcare needs and technological advancements (13, 19–26).

## 5 RPM in Canadian healthcare: benefits and limitations

The increased adoption of RPM in Canada, particularly after March 2023, has introduced both substantial benefits and notable challenges within the healthcare system (13, 19–26).

### 5.1 Improved accessibility

RPM expands access to healthcare, particularly for remote and underserved communities, effectively bridging geographical and logistical gaps. By enabling timely monitoring and care, RPM improves healthcare accessibility for populations with limited access to in-person medical services (13, 19–26).

### 5.2 Cost reduction

RPM's capacity to prevent hospital readmissions and reduce emergency visits contributes to lower healthcare expenses, making it a cost-effective solution within Canada's publicly funded healthcare model. The Canadian Institute for Health Information (CIHI) highlights RPM's role in enhancing healthcare efficiency, particularly valuable in a system facing budget constraints and an aging population (13, 19–26).

### 5.3 Technological progress

RPM has spurred significant innovation in healthcare technology, catalyzing the development of more sophisticated tools for patient monitoring, data analytics, and predictive modeling. These advancements continue to enhance healthcare delivery and patient outcomes by enabling more personalized and data-driven care approaches (13, 19–26).

### 5.4 Loss of human contact

While RPM enhances efficiency, it may reduce personal interaction between patients and healthcare providers. Many patients, particularly those with chronic conditions, highly value regular in-person contact and may feel isolated or less engaged when most interactions occur digitally. This shift highlights a potential drawback of RPM, where the convenience of remote care may come at the cost of the personal connections essential to holistic care (13, 19–26).

### 5.5 Data security

Privacy concerns related to data security remain a critical challenge, potentially limiting RPM adoption. Patients may hesitate to participate in RPM programs if they perceive a risk to their personal health information, underlining the need for stringent data protection measures to build and maintain trust (13, 19–26).

### 5.6 Digital divide

Access to RPM remains uneven across Canada, especially in remote areas with limited internet connectivity. This disparity contributes to a digital divide, where patients in digitally underserved regions cannot fully benefit from RPM technologies. Addressing this gap is essential to ensuring equity in healthcare access and preventing RPM from unintentionally widening healthcare disparities (13, 19–26).

These benefits and limitations illustrate RPM's dual impact on Canadian healthcare. While RPM offers a promising avenue for improving access, reducing costs, and driving technological progress, overcoming challenges related to patient-provider interaction, data security, and equitable access is essential. Addressing these limitations will be critical to maximizing RPM's positive impact and achieving a balanced integration within Canada's healthcare landscape (13, 14).

## 6 Integration of AI in remote patient monitoring (RPM)

### 6.1 Enhancing RPM and shaping future care delivery

Integrating Artificial Intelligence (AI) into healthcare has emerged as a transformative force with substantial implications for the future of Remote Patient Monitoring (RPM). AI's ability to analyze large volumes of patient data and identify patterns swiftly holds significant potential for enhancing RPM's effectiveness and expanding its capabilities. Leveraging machine learning algorithms, AI can detect early warning signs in chronic disease patients, enabling proactive interventions that could reduce hospitalizations and improve health outcomes. For example, AI-driven predictive models in RPM platforms analyze real-time vital signs, generating alerts for healthcare providers when data suggests a heightened risk of complications (27–33).

AI applications in healthcare also extend to natural language processing, which supports virtual consultations, and image recognition, which aids diagnostics, both valuable complements to RPM. AI's capacity for personalization allows it to adjust monitoring parameters based on individual patient history and preferences, promoting a more patient-centred approach. This tailored monitoring aligns with the evolving healthcare paradigm, which emphasizes individualized care and prevention (27–33).

To support the ethical and effective integration of AI, healthcare organizations could benefit from an AI maturity assessment tool that evaluates and tracks progress in AI adoption. Such a tool would ensure that AI applications align with ethical standards, safeguard accessibility, and foster transparency. It could also enable healthcare providers to monitor AI's development, helping establish fair, unbiased, and secure practices that enhance, rather than hinder, healthcare access (27–33).

As healthcare delivery shifts toward preventive and community-based models, AI is poised to play a pivotal role in making RPM systems more autonomous and accessible, particularly in underserved areas. However, realizing AI's full potential in RPM requires ongoing attention to ethical data usage, transparency in AI decision-making, and robust data security protocols. Addressing these considerations is crucial for maintaining patient trust and ensuring that AI advancements contribute positively to healthcare (1–3, 12–14).

The integration of AI with RPM represents a promising pathway toward a healthcare system that is more responsive, efficient, and adaptable to the diverse needs of the Canadian population. By facilitating early interventions and supporting personalized care, AI-enhanced RPM can help shape a future healthcare landscape that is not only technologically advanced but also deeply attuned to the principles of equitable, patient-centred care.

### 6.2 Readiness, limitations, and regulatory concerns

The potential of Artificial Intelligence (AI) to enhance Remote Patient Monitoring (RPM) is immense, particularly in areas like predictive analytics, personalized care, and early intervention for chronic conditions. However, the current readiness of AI to seamlessly integrate with RPM technologies is still evolving, and several challenges remain. Despite significant advancements in AI, the technology is not yet fully capable of delivering consistent, real-time, and accurate predictions across all healthcare settings. Issues such as data quality, algorithmic bias, and the need for large, high-quality datasets hinder AI's broad application in RPM systems.

Additionally, regulatory and bioethical concerns surrounding AI in healthcare must be addressed before its widespread implementation in RPM. Regulatory frameworks, such as those enforced by Health Canada and the U.S. FDA, are still catching up with AI technologies. The lack of standardized guidelines for AI integration into healthcare platforms presents challenges for approval and deployment. Bioethically, concerns about patient consent, data privacy, and the transparency of AI decision-making remain critical. There is a need for clear policies that govern AI's ethical use, ensuring that patient data is protected, and that AI algorithms can be audited for accuracy and fairness.

While AI holds significant promise for RPM, its integration into existing systems must be approached cautiously, with attention to its readiness, the regulatory environment, and ethical considerations. Ensuring AI's responsible and effective use will require ongoing collaboration between healthcare providers, technologists, and regulatory bodies.

## 7 Future and global perspectives

### 7.1 The evolving role of RPM in Canadian healthcare

Looking ahead, RPM is set to solidify its role as an integral component of healthcare delivery in Canada. Several key areas warrant attention to ensure RPM's successful integration and sustainability, including enhanced interoperability, strengthened data security, expanded accessibility, and ongoing education for both patients and providers (1–4, 34, 35).

### 7.2 Standardizing interoperability protocols

With the growing adoption of RPM, establishing standardized data-sharing frameworks between RPM platforms and Electronic Medical Records (EMRs) is essential. Developing interoperability standards will facilitate seamless integration of RPM data into clinical workflows, enhancing continuity of care and supporting evidence-based decision-making. Such standardization could enable RPM to function as a cohesive part of the broader healthcare ecosystem (1–4, 32–35).

### 7.3 Enhancing data privacy and security

Given the sensitive nature of health data, advancing encryption technologies and implementing stringent cybersecurity protocols will be paramount. Compliance with regulations like PIPEDA and PHIPA is essential to building patient trust and ensuring secure data handling. Enhanced data security measures could encourage more patients and providers to adopt RPM, with the confidence that their information is protected (1–4, 12–14, 32–35).

### 7.4 Expanding accessibility and addressing the digital divide

To ensure equitable access to RPM, efforts to improve digital infrastructure in underserved areas are crucial (13, 14). Investments from both the government and private sector in expanding high-speed internet access to remote communities would enable more patients to benefit from RPM's preventive capabilities. Bridging the digital divide is essential for creating a more inclusive healthcare system that fully leverages RPM's potential (1–4, 12–14, 32–35).

### 7.5 Enhancing patient and provider education

Educating both patients and healthcare providers about RPM's benefits, functionalities, and usage can help build digital literacy and optimize RPM's impact (13, 14). By increasing comfort and awareness around RPM, patients are more likely to engage actively in their healthcare, while providers can use RPM more effectively as part of patient care strategies, ultimately leading to improved outcomes (1–4, 12–14, 32–35).

### 7.6 Innovating in real-time analytics and AI integration

As RPM technology continues to evolve, integrating artificial intelligence (AI) and real-time data analytics offers the potential to enhance RPM's predictive capabilities further. These advancements could allow providers to identify early signs of health deterioration, enabling interventions before symptoms become more severe. The fusion of AI with RPM has the potential to shift healthcare towards a model focused on preemptive, precision care (1–4, 12–14, 32–35).

In summary, RPM is positioned to become an increasingly vital element of Canadian healthcare. Transitioning to a healthcare model that leverages RPM for early detection, proactive care, and patient engagement could significantly enhance health outcomes, increase accessibility, and address several of Canada's most pressing healthcare challenges. With focused efforts in these areas, RPM has the potential to transform healthcare delivery, contributing to a more responsive, inclusive, and efficient system.

### 7.7 Patient-centered design and user experience in RPM

The successful integration of Remote Patient Monitoring (RPM) depends not only on the underlying technology but also on how well these solutions are tailored to the needs and preferences of patients. Patient-centred design and user experience (UX) are critical components that influence the adoption and effectiveness of RPM platforms (36–38).

Patient-centered design involves creating systems and interfaces that are intuitive, accessible, and aligned with the capabilities and expectations of end-users, which is particularly important for diverse populations, including the elderly and individuals with low digital literacy. Well-designed RPM UX can increase treatment adherence, improve patient satisfaction, and promote better health outcomes (36–38).

However, there are UX challenges in RPM that need to be addressed. Interface complexity can discourage the continuous use of RPM, making it essential to simplify navigation and provide clear instructions. Ensuring that platforms are accessible to people with disabilities is fundamental, including options for adjustable font sizes, compatibility with screen readers, and design considerations for color blindness. Additionally, interactive features such as personalized reminders, real-time feedback, and gamification can increase patient engagement with RPM (36–38).

To improve the user experience, strategies like co-creation with patients can result in more effective and user-friendly platforms (13, 14, 39–41). Conducting rigorous usability testing helps identify and resolve issues before large-scale implementation. Providing educational resources and technical support assists patients in becoming familiar with RPM platforms, increasing confidence and proficiency in using the technology (39–41).

A positive user experience is fundamental for sustained RPM adoption. Platforms that meet patient needs not only improve satisfaction but also increase the likelihood of positive health outcomes due to greater adherence and engagement (39–41). Therefore, investing in patient-centred design and optimizing the user experience is essential to maximize RPM's impact on improving healthcare in Canada.

### 7.8 Global trends in remote patient monitoring (RPM) and Canada's role in driving innovation

Remote Patient Monitoring (RPM) is rapidly expanding globally, with varying levels of adoption across regions, influenced by economic, technological, and healthcare system factors. In the United States and European Union, RPM has been increasingly integrated into healthcare systems, particularly for managing chronic diseases and reducing hospital readmissions. The U.S. has seen substantial adoption due to favorable regulatory changes such as reimbursement policies for RPM services under Medicare and Medicaid. Similarly, in the EU, RPM adoption has been accelerated by initiatives such as the European Health Data Space (EHDS), which facilitates data sharing across member states and enhances the scalability of digital health interventions.

However, challenges persist globally, including disparities in access to technology, digital literacy, and regulatory frameworks. For example, low- and middle-income countries face significant barriers to widespread RPM adoption, including inadequate digital infrastructure and limited financial resources for healthcare technologies. In these regions, mobile health (mHealth) applications are often seen as a potential solution, but the integration of RPM systems remains constrained.

Canada, with its strong healthcare infrastructure and universal health system, has positioned itself as a leader in RPM adoption. Canada's experience in deploying RPM technologies across its diverse geography, including rural and remote communities, provides a valuable model for other countries. The country's efforts to tackle challenges like interoperability, data privacy, and regulatory compliance serve as a blueprint for others to follow. Furthermore, as Canada's RPM ecosystem continues to expand, the lessons learned from its integration of AI, real-time monitoring, and patient engagement could offer significant insights for global healthcare systems seeking to enhance care delivery.

Globally, Canada stands out in fostering collaboration between public and private sectors to drive the evolution of RPM technologies. The insights from Canadian healthcare providers, regulators, and researchers could help accelerate RPM integration in other countries, particularly in addressing regulatory barriers, improving system interoperability, and ensuring equitable access for underserved populations.

As RPM technologies continue to evolve, the global community can benefit from Canada's approach by leveraging its successful integration strategies, while Canada, in turn, can learn from the global expansion of RPM to further refine its own healthcare models. The mutual exchange of knowledge, expertise, and technological advancements promises to enhance healthcare delivery worldwide.

## 8 Conclusion

Integrating Remote Patient Monitoring (RPM) into Canadian healthcare marks a significant shift toward more accessible, efficient, and patient-centered care. Platforms like TELUS Health, Cloud DX, and McKesson Canada have illustrated RPM's effectiveness in managing chronic diseases, reducing hospital readmissions, and extending healthcare services to remote and underserved communities. Although RPM faces challenges—such as data privacy concerns, regulatory complexities, and the digital divide, these obstacles are not insurmountable. Addressing these issues will be essential to ensuring RPM's continued growth and broader adoption across Canada.

Future advancements in RPM are expected to enhance its impact further, especially as improvements in interoperability, data security, and digital accessibility are realized. By embedding RPM more comprehensively into healthcare delivery, Canada has the opportunity to foster a resilient, inclusive, and preventive healthcare system. Integrating Artificial Intelligence (AI) with RPM represents an exciting direction, as AI's predictive, personalized, and real-time analytics capabilities can amplify RPM's impact on patient outcomes. However, responsible AI integration will require adopting an AI maturity assessment framework to maintain alignment with ethical standards and support equitable healthcare access.

## Data Availability

The original contributions presented in the study are included in the article/Supplementary Material, further inquiries can be directed to the corresponding author/s.
